# Postmortem Toxicology of New Synthetic Opioids

**DOI:** 10.3389/fphar.2018.01210

**Published:** 2018-10-26

**Authors:** Marta Concheiro, Rachel Chesser, Justine Pardi, Gail Cooper

**Affiliations:** ^1^Department of Sciences, John Jay College of Criminal Justice, City University of New York, New York, NY, United States; ^2^Department of Forensic Toxicology, New York Office of the Chief Medical Examiner, New York, NY, United States

**Keywords:** opioids, synthetic opioids, fentanyl, postmortem toxicology, blood

## Abstract

One hundred fifteen Americans die every day from opioid overdose. These overdose fatalities have been augmented by the increased availability of potent synthetic opioids, such as fentanyl and its derivatives. The death rate of synthetic opioids, other than methadone, increased by 72.2% from 2014 to 2015, and doubled from 2015 to 2016, situating the USA in the midst of an opioid overdose epidemic. The analytical identification of these opioids in postmortem samples and the correct toxicological data interpretation is critical to identify and implement preventive strategies. This article reviews the current knowledge of postmortem toxicology of synthetic opioids and the chemical and pharmacological factors that may affect drug concentrations in the different postmortem matrices and therefore, their interpretation. These factors include key chemical properties, essential pharmacokinetics parameters (metabolism), postmortem redistribution and stability data in postmortem samples. Range and ratios of concentrations reported in traditional and non-traditional postmortem specimens, blood, urine, vitreous humor, liver and brain, are summarized in tables. The review is focused on fentanyl and derivatives (e.g., acetyl fentanyl, butyryl fentanyl, carfentanil, furanyl fentanyl, 4-methoxybutyrylfentanyl, 4-fluorobutyrylfentanyl, ocfentanil) and non-traditional opioid agonists (e.g., AH-7921, MT-45, U-47700). All of these data are critically compared to postmortem data, and chemical and pharmacological properties of natural opioids (morphine), semi-synthetic (oxycodone, hydrocodone, hydromorphone, and oxymorphone), and synthetic opioids (methadone and buprenorphine). The interpretation of drug intoxication in death investigation is based on the available published literature. This review serves to facilitate the evaluation of cases where synthetic opioids may be implicated in a fatality through the critical review of peer reviewed published case reports and research articles.

## Introduction

Opioid overdose deaths continue to increase in the United States, killing more than 42,000 people in 2016. The opioids detected in these cases, in increasing order, were methadone, natural and semi-synthetic opioids (e.g., oxycodone, hydrocodone), heroin and synthetic opioids (e.g., fentanyl, fentanyl-analogs). Synthetic opioids (excluding methadone) and heroin deaths specifically experienced a sharp increase from 2015 to 2016 (20 and 100%, respectively) (Seth et al., 2018). Fentanyl and its derivatives have been increasingly present as adulterants mainly in heroin, but also in other drugs such as cocaine and synthetic cannabinoids (Coopman and Cordonnier, 2017; Armenian et al., 2018), due to their ease of manufacturing and readily available precursors shipped from China (Armenian et al., 2018). In addition to being present in other drugs supply, fentanyl analogs have been also marketed as “research chemicals” and can easily be acquired over the internet. Due to their high potency and the increased use of heroin as an initiating opioid of abuse (8.7% in 2005 vs. 33.3% users in 2015) (Cicero et al., 2017; O'Donnell et al., 2017), the number of opioid-related deaths have drastically increased in the recent years. Given that opioid novices have limited tolerance to opioids, a slight imprecision in dosing inherent in heroin use and/or the presence of potent fentanyl and analogs, can be fatal.

Fentanyl, its analogs (e.g., acetyl fentanyl, 3-methylfentanyl, alphamethylfentanyl, furanyl fentanyl) and the new generation synthetic opioids (e.g., AH-7921, U-47700, MT-45) have a chemical core structure totally different from morphine, a naturally occurring opioid from *Papaver somniferum* and reference compound of the opioids group; but all of them act on the opioid receptor (mu-receptor) reducing the intensity of pain and showing a high addiction potential. These opioid receptor agonists also induce dose-dependent respiratory depression (Pattinson, 2008), which is the main reason for their life-threatening risk (Ujváry et al., 2017). Fentanyl is approximately 200 times more potent than morphine, and the potencies of its analogs are variable, from 7 times more potent than morphine for butyrfentanyl and furanyl fentanyl, to more than 4,000 and 10,000 times for sufentanil and carfentanil, respectively (UNODC, 2017). The new generation opioids AH-7921 and MT-45 show similar potency to morphine (Brittain et al., 1977; EMCDDA, 2015), and U-47700 about 7.5 times more potent (Cheney et al., 1985).

Synthetic opioids are widely regulated by the United States Controlled Substances Act of 1970 (CSA) in order to control their use and distribution. As new compounds arise and threaten public safety, compounds can be emergency scheduled by the DEA to slow production and use of these harmful substances and aid in prosecution of drug diverters for a temporary period until the formal procedures have gone through (US Drug Enforcement Administration, 2017). Substances are classified into schedules in the CSA based on their safety, medicinal use and potential for abuse. A Schedule I substance is classified as having no currently accepted medical use and a high abuse potential. Examples of synthetic opioids in Schedule I include furanyl fentanyl, U-47700, acetyl fentanyl and 3-methyl fentanyl. Schedule II classified opioids have a high potential for abuse but have current medicinal uses like fentanyl which is used as an anesthetic and analgesic, as well as carfentanil, remifentanil and sufentanil (US Drug Enforcement Administration, 2017). Most recently, the DEA issued a temporary scheduling order for all fentanyl –related substances (to include all analog modifications) in February of 2018, which cover all substances that were not already classified into Schedule I of the CSA in an aggressive attempt to regulate the manufacture and subsequent trafficking of new synthetic opioids into the United States (Drug Enforcement Administration, 2018).

The expansion of these new synthetic opioids constitutes an important challenge in forensic toxicology. First of all, most of these substances are not detected in the routine screening and confirmation methods in the laboratory. Also, due to the low doses employed of these highly potent drugs, the concentrations expected in the biological samples are in the low ng to pg/mL or ng to pg/g range, requiring extremely sensitive methods of analysis. Recently, Marchei et al. (2018) and Liu et al. (2018) reviewed the currently available screening and confirmation methods of new synthetic opioids in biological and non-biological samples. As indicated by Marchei et al. (2018), gas chromatography combined with mass spectrometry (GC-MS) and more frequently liquid chromatography tandem mass spectrometry (LC-MSMS) are the most common techniques due to their sensitivity and specificity. However, given the continued development of new derivatives, the major disadvantage of these target techniques, which employ quadrupole mass spectrometers, is that are limited by the reference standards available. High resolution mass spectrometry (time-of-flight, orbitrap) offers potential advantages to identify unknown compounds without the availability of a reference standard, but this technology is not readily available in most forensic laboratories (Marchei et al., 2018).

Regarding biological samples, most of these methods have been developed in blood or urine, and the target analytes are the parent compounds and rarely the metabolites (Marchei et al., 2018). In postmortem toxicology, other biological specimens such as vitreous humor, liver and brain are commonly analyzed. Unfortunately, fully validated methods for the determination of synthetic opioids in these specimens are lacking in the literature. This is in part due to the constant changes in illicit synthetic opioids being identified and laboratories being unable to justify the extensive time and cost associated with fully validating a method for a drug that may only be present in cases for a short time. Analytical methods in forensic toxicology are commonly validated in the corresponding biological sample following the guidelines published by the Scientific Working Group in Forensic Toxicology (SWGTOX) (Scientific Working Group for Forensic Toxicology, 2013) to guarantee the analytical quality of the measured concentrations. The analysis of metabolites in the different biological matrices may improve the interpretation of the results, extending the detection window and indicating if it was an acute or a delayed-death evaluating the metabolite-to-parent ratios. Recent publications about the identification of new metabolites of the synthetic opioids are available (Wohlfarth et al., 2016; Steuer et al., 2017; Watanabe et al., 2017; Krotulski et al., 2018a); however, its application to authentic samples is still scarce (Poklis et al., 2015; Staeheli et al., 2016; Martucci et al., 2017; Allibe et al., 2018).

Besides the analytical challenges associated with synthetic opioids, due to the scarcity of available postmortem data, the interpretation of the results is extremely difficult. Conducting postmortem toxicology interpretation provides a number of very significant challenges to the forensic toxicologist. The range of postmortem specimens (blood, urine, vitreous humor, tissues, hair), the lack of reference databases, the presence of other substances (e.g., benzodiazepines, alcohol), opioid tolerance, and postmortem phenomena (postmortem redistribution and drug instability) complicates the interpretation of the analytical findings. Pichini et al. (2018) and Zawilska (2017) discussed non-fatal and lethal intoxications involving the new synthetic opioids, and Drummer (2018) focused his review on fatalities due to these compounds.

The present review is focused on fentanyl derivatives and new generation opioids due to the limited knowledge concerning these substances and their high prevalence in opioid-overdose related cases. This work complements the previously published literature reviewing the current knowledge of postmortem toxicology of synthetic opioids and the chemical and pharmacological factors that may affect drug concentrations in the different matrices and therefore, their interpretation in postmortem samples. These factors include key chemical properties, essential pharmacokinetics parameters, postmortem redistribution and stability data in postmortem samples. All of these data are critically compared to postmortem data of natural opioids (morphine), semi-synthetic (oxycodone, hydrocodone, hydromorphone, and oxymorphone), and synthetic opioids (methadone and buprenorphine). The interpretation of drug intoxication in death investigation is based on the available published literature. This review serves to facilitate the evaluation of cases where synthetic opioids may be implicated in a fatality through the review of peer reviewed published case reports and research articles.

## Methods

PubMed, Scopus and Google Scholar were searched for appropriate articles. Forensic case-reports and research articles of natural, semi-synthetic and synthetic opioids were reviewed up to May 2018. All articles were manually reviewed for content and references in each manuscript were further queried. Included articles were limited to peer-reviewed journals indexed by the Institute for Scientific Information (ISI) and published in English. Chemical properties were retrieved from the public databases PubChem (https://pubchem.ncbi.nlm.nih.gov/) and DrugBank (https://www.drugbank.ca/drugs).

## Chemical and pharmacological properties

**Figure 1:**
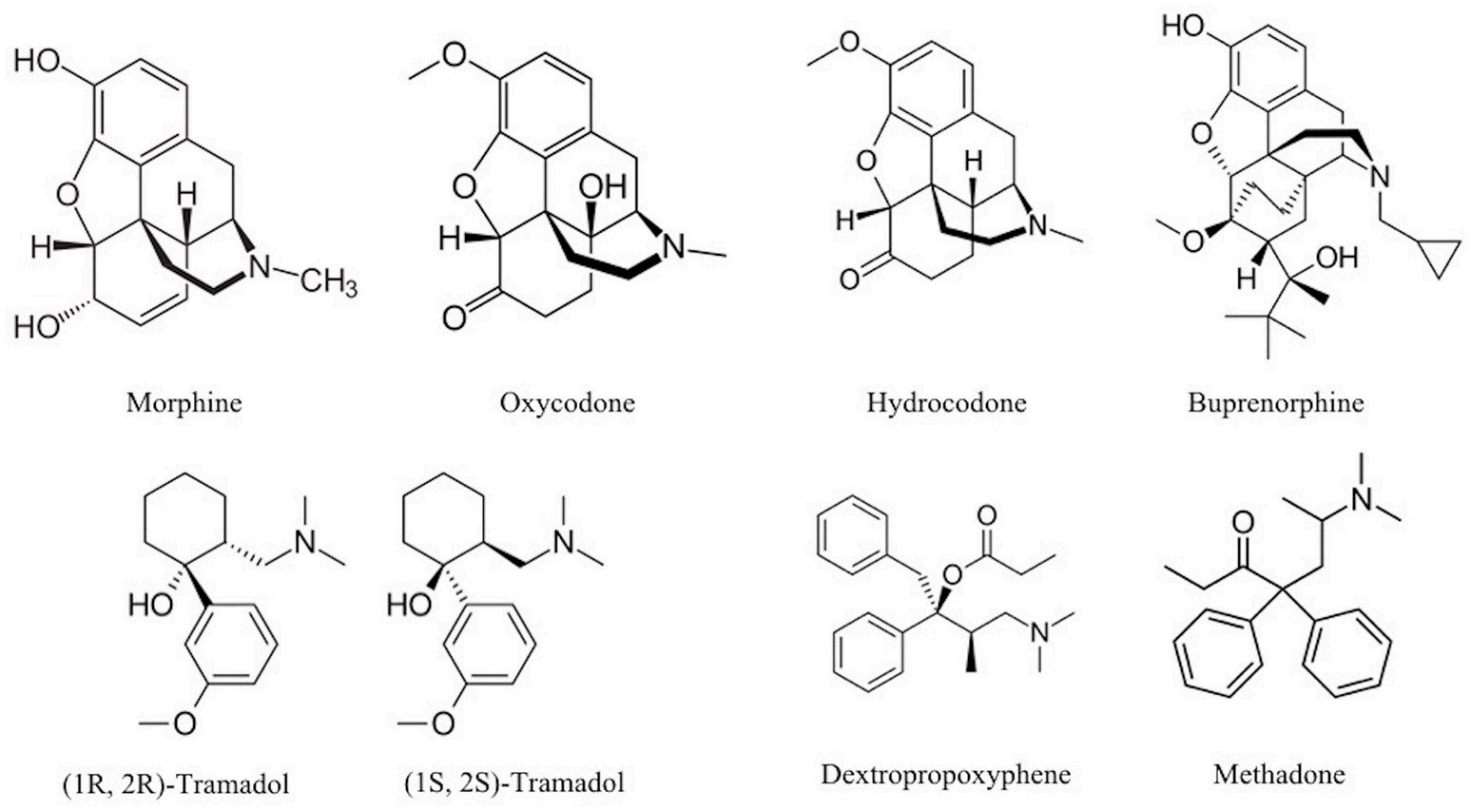
Chemical structures of selected classic opioids.

**Figure 2:**
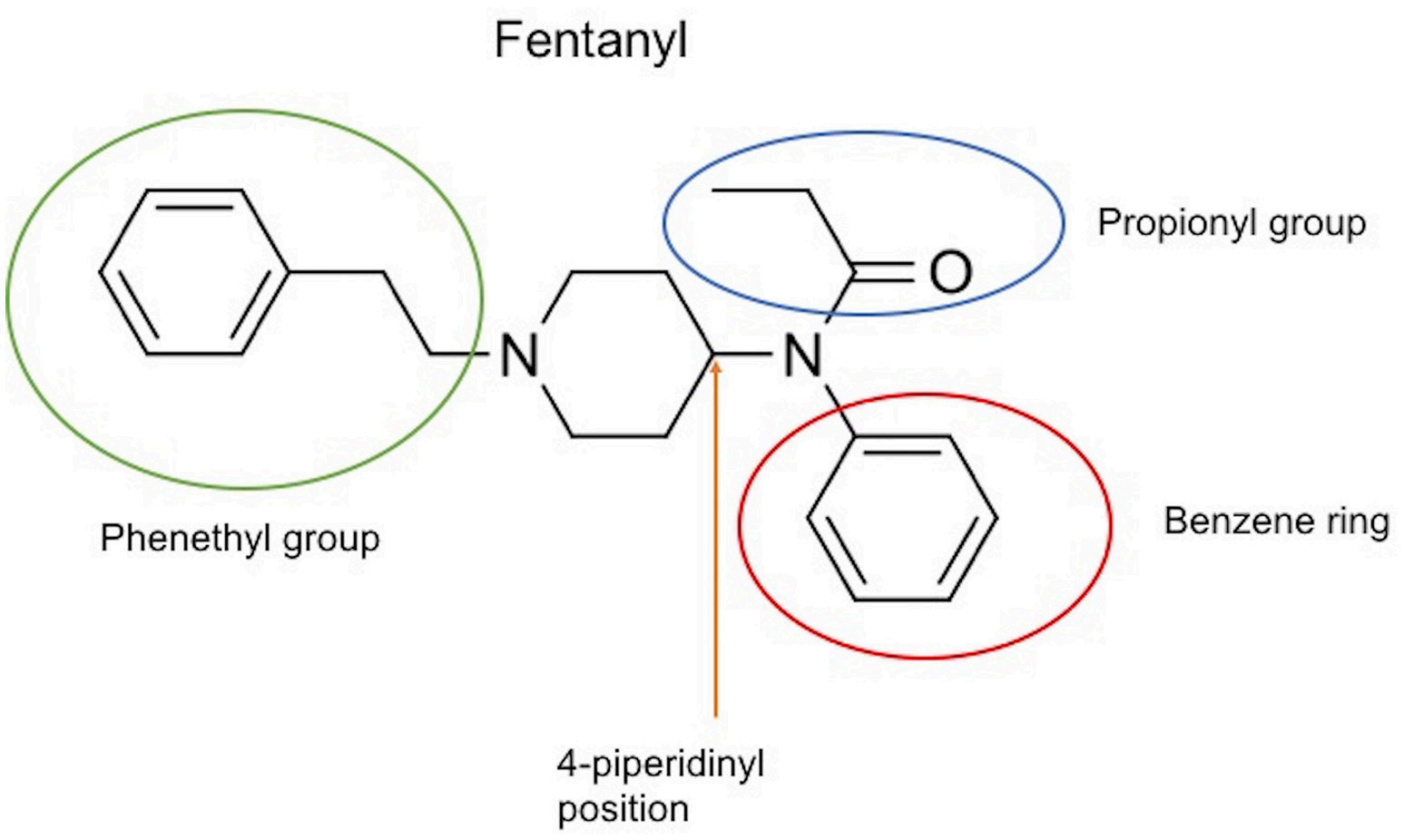
Chemical structure of fentanyl.

**Figure 3:**
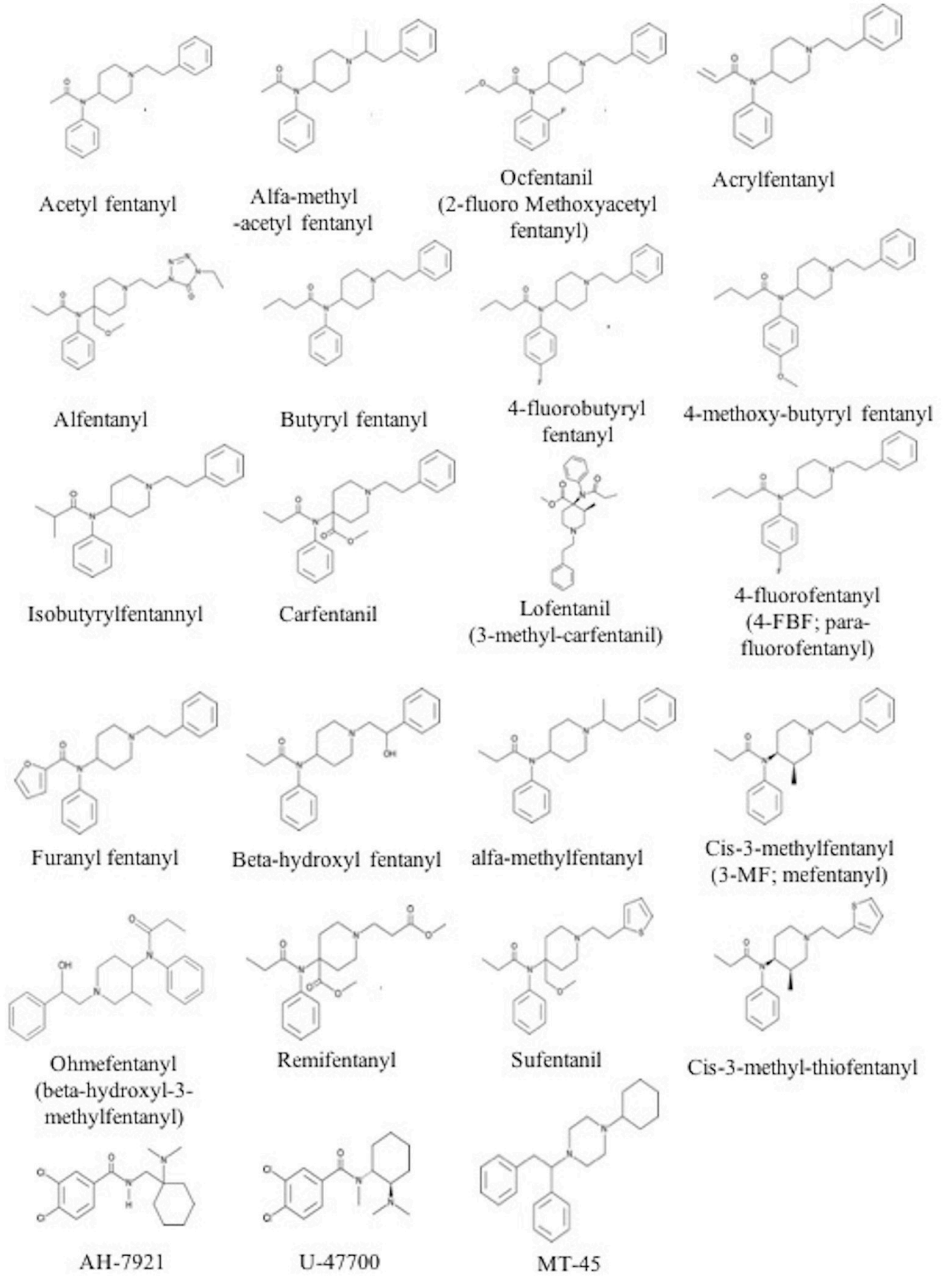
Chemical structures of 20 fentanyl derivatives and 3 new generation opioids not related to fentanyl.

The chemical structure of the diverse synthetic opioids, including fentanyl and analogs, differs significally from the chemical structure of morphine and semi-synthetic opioids (e.g., oxycodone, hydrocodone, buprenorphine). Figure 1 summarizes the chemical structure of selected classic opioids. Fentanyl is a piperidinyl derivative with moieties on the nitrogen and the 4-position (Figure 2). The different fentanyl derivatives show substitutions on the propionyl moiety (e.g., acetylfentanyl, acrylfentanyl, butyrfentanyl, furanyl fentanyl), phenethyl moiety (e.g., ohmefentanyl), N-phenyl ring (e.g., ocfentanil, 4-methoxy-butyrylfentanyl) and/or at the 4-piperidinyl-position (e.g., carfentanil). The chemical structures of the new generation synthetic opioids (AH-7921, U-47700, MT-45) are different from fentanyl. Figure 3 shows 20 fentanyl derivatives and 3 new generation synthetic opioids not related to fentanyl. Due to the close chemical structure among fentanyl derivatives, some compounds, such as cyclopropyl fentanyl and crotonyl fentanyl, have exactly the same molecular formula, and therefore, the same molecular weight. As a consequence of this, special attention has to be paid in the development of the analytical methods for the determination of these compounds, and a complete chromatographic separation is required to guarantee their correct identification by gas or liquid chromatography coupled to mass spectrometry (GC-MS, LC-MSMS).

Chemically, opioids are predominantly basic drugs with pKa ranging from 7.5 to 10.9. The chemical parameter log P, the decimal logarithm of the partition coefficient Kp, is a useful indication of the lipophilicity of a compound. In the case of opioids, log P range is wide, from 0.8 (oxymorphone) to 5 (methadone). Morphine and related compounds show the lowest log *P* values (0.8–2). Fentanyl and analogs show a log P between 1.5 and 4.3. The high lipophilicity of fentanyl and its analogs enables rapid diffusion through membranes, including the blood-brain barrier. Also, this lipophilicity along with their basic characteristics make these group of drugs candidates to undergo postmortem redistribution. Table 1 summarizes the molecular weight, pKa and log P of selected opioids.

**Table 1:** Monoisotopic molecular weight (g/mol), pKa and Log P of selected natural, semi-synthetic and synthetic opioids.

**Group**	**Analyte**	**Monoisotipic molecular weight (g/mol)**	**pKa**	**Log P**
Natural and semi-synthetic opioids	Morphine	285.136	8.2	0.9
	Codeine	299.152	9.2	1.3
	Hydrocodone	299.152	8.6	2.0
	Hydromorphone	285.133	8.6	1.6
	Oxycodone	315.147	8.2	1.0
	Oxymorphone	301.131	10.9	0.8
	Buprenorphine	467.300	7.5	4.5
Synthetic opioids	Fentanyl	336.220	8.8	3.8
	Methadone	309.445	9.1	5.0
	Tramadol	263.189	9.2	2.5
Synthetic opioids-Fentanyl derivatives	alphamethylacetylfentanyl; acetyl-alpha-methylfentanyl	336.220	9.01	3.5
	Alfentanil	416.253	7.5	2.8
	Butyryl fentanyl; butyr fentanyl	350.235	8.77	4.3
	Carfentanil	394.225	8.05	3.7
	3-methylcarfentanil; lofentanil	408.241	8.36	4.2
	4-fluorofentanyl; 4-FBF; para-fluorofentanyl	354.210	8.74	4.0
	beta-hydroxyfentanyl	352.215	8.28	2.9
	alpha-methylfentanyl	350.235	9	4.2
	cis-3-methylfentanyl; 3-MF; mefentanyl	350.235	9.08	4.3
	beta-hydroxy-3-methylfentanyl; ohmefentanyl	366.230	8.59	3.4
	Remifentanil	376.199	7.51	1.5
	Sufentanil	386.202	8.86	3.6
	3-methylthiofentanyl	356.192	9.07	4.2

Volume of distribution (Vd) and protein binding also help to predict the drugs that may exhibit postmortem redistribution. Vd is defined as the volume into which the total amount of the drug would have to be uniformly distributed to reach the concentrations measured in plasma. It is expressed in L/kg of body weight (amount of drug in the body divided by the plasma drug concentration). Drugs highly bound to plasma proteins but not to tissue components would be expected to have a small Vd, while those drugs which distribute into muscle, adipose tissue and other intracellular components will have a high Vd. Drugs with a Vd greater than 3 L/kg are considered to have a greater potential to undergo postmortem redistribution. Table 2 summarizes the Vd and protein binding data currently available for selected opioids.

**Table 2:** Critical pharmacological properties in postmortem toxicology, volume of distributon (Vd), protein bining and potency relative to morphine, of selected natural, semi-synthetic and synthetic opioids.

**Group**	**Analyte**	**Vd (L/kg)**	**Protein binding (%)**	**Potency relative to morphine**	**References**
Natural and semi-synthetic opioids	Morphine	1–6	30–40	1	Baselt, 2017
	Codeine	2.5–3.5	7–25	0.3	Baselt, 2017
	Hydrocodone	3.3–4.7	19–45	0.5–1	Patanwala et al., 2007; Baselt, 2017
	Hydromorphone	2.9	20	5–10	Bruera et al., 1996; Patanwala et al., 2007; Baselt, 2017
	Oxycodone	2.6	45	1	Patanwala et al., 2007; Al-Asmari et al., 2009
	Oxymorphone	3	10–12	10	Patanwala et al., 2007; Smith, 2009
	Buprenorphine	3–5	96	40	Dahan et al., 2005
Synthetic opioids	Fentanyl	3–8	80–85	224	Jumbelic, 2010
	Methadone	1–8	85–90	3–5	Patanwala et al., 2007; Baselt, 2017
	Tramadol	3	20	0.1	Christoph et al., 2007; Oertel et al., 2011
Synthetic opioids-Fentanyl derivatives	Acetylfentanyl	NA	NA	15	Higashikawa and Suzuki, 2008
	Acrylfentanil	NA	NA	170	Ujváry et al., 2017
	Alfentanil	0.4–1	92	72	Vardanyan and Hruby, 2014
	Butyryl fentanyl; butyr fentanyl	NA	NA	7	Higashikawa and Suzuki, 2008
	Isobutyrylfentanyl	NA	NA	1.3–6.9	Higashikawa and Suzuki, 2008
	Carfentanil	NA	NA	10,000	Van Bever et al., 1976
	Furanyl fentanyl	NA	NA	7	Higashikawa and Suzuki, 2008
	alpha-methylfentanyl	NA	NA	56.9	Higashikawa and Suzuki, 2008
	cis-3-methylfentanyl; 3-MF; mefentanyl	NA	NA	6000	Higashikawa and Suzuki, 2008
	Remifentanil	0.35	70	220	Wax et al., 2003
	Sufentanil	NA	NA	4,520	Niemegeers et al., 1976
Synthetic opioids-Not related to fentanyl	AH-7921	NA	NA	1	Hayes and Tyers, 1983
	U-47700	NA	NA	7.5	Cheney et al., 1985
	MT-45	NA	NA	1	EMCDDA, 2015

*NA, not available*.

One of the critical issues related to fentanyl, its derivatives and the new synthetic opioids, is the low concentrations expected in the biological samples (ng to pg/mL or ng to pg/g range) due to their high potency. However, the potency of these type of drugs varies considerably within this group, and therefore the concentrations reported show a wide range, depending on the drug. Table 2 summarizes the potencies relative to morphine for selected opioids.

## Metabolism

The identification and quantification of metabolites in postmortem samples may improve the interpretation of the analytical results. The determination of metabolites may extend the window of detection, and also can be employed to calculate metabolite-to-parent ratios in urine and other biological samples to differentiate acute or delayed death. In certain cases, as it happens in morphine and buprenorphine, metabolites can be pharmacologically active. Although this type of information is limited in the case of the synthetic opioids, fentanyl, sufentanil, and alfentanil's metabolites are inactive in the opioid system (Schneider and Brune, 1986).

Although the utility of metabolite determination in biological samples is known, its application to authentic specimens is still scarce in the case of synthetic opioids due to the limited data available about their metabolism (Poklis et al., 2015; Staeheli et al., 2016; Martucci et al., 2017; Allibe et al., 2018). Recent publications about the identification of new metabolites of the synthetic opioids *in vivo* and *in vitro* are available (Wohlfarth et al., 2016; Steuer et al., 2017; Watanabe et al., 2017; Krotulski et al., 2018a). While *in vitro* studies utilizing human liver hepatocytes or microsomes can identify multiple primary and secondary metabolites for a particular fentanyl derivative, actual human specimens typically show lower number and/or a different metabolite prevalence profile, so studies investigating the presence of the *in vitro* metabolites in authentic human samples are highly encouraged. Table 3 summarizes recent publications about the identification of new metabolites of synthetic opioids *in vitro* and *in vivo*.

**Table 3:** *In vitro* and *in vivo* metabolism of synthetic opioids.

**Compound**	**Study type**	**Matrix (species)**	**Total # phase I metabolites**	**Major metabolites (decreasing order of relative intensity)**	**Phase II metabolites**	**Recommended target analytes in urine**	**References**
Acetyl Fentanyl 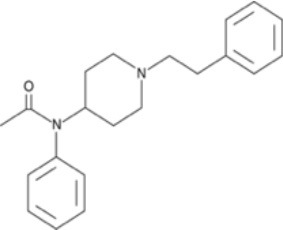	*In vivo*	Urine (humans)	6	– Hydroxylated metabolite at phenylethyl ring – Hydroxy-methoxy metabolite at phenylethyl ring	Glucuronide of hydroxylated metabolites	– Hydroxylated metabolite at phenylethyl ring – Hydroxy-methoxy metabolite at phenylethyl ring – Acetyl fentanyl	Melent'ev et al., 2015
	*In vitro*	Pool human liver hepatocytes	7	– N-dealkylated metabolite at the piperidine moiety – Hydroxylated metabolites at the ethyl linker – Dihydroxylation at phenylethyl ring			Watanabe et al., 2017
	*In vivo*	Urine (human)	24	– Hydroxy-methoxy metabolite at phenylethyl ring – Hydroxy metabolite at the ethyl linker – N-dealkylated metabolite at the piperidine moiety	Glucuronides and sulfates of hydroxy-metabolites	– Hydroxy metabolite at the ethyl linker – Hydroxy-methoxy metabolite at phenylethyl ring – Acetyl fentanyl	
	*In vitro*	Pluripotent stem cell-derived hepatocytes	6	– N-dealkylated metabolite at the piperidine moiety – Hydroxylated metabolite at phenylethyl ring – Hydroxylated metabolites at the ethyl linker			Kanamori et al., 2018
Acrylfentanyl 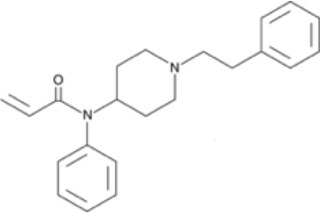	*In vitro*	Pool human liver hepatocytes	8	– N-dealkylated metabolite at the piperidine moiety – Hydroxylated metabolite at the piperidine moiety – Hydroxylated metabolite at the ethyl linker			Watanabe et al., 2017
	*In vivo*	Urine (human)	12	– N-dealkylated metabolite the piperidine moiety – Hydroxylated at the ethyl linker – Dihydroxylated metabolite at the piperidine and at the ethyl linker – Hydroxy-methoxy metabolite at phenylethyl ring	Glucuronides of hydroxy-metabolites	– Hydroxylated at the ethyl linker – Dihydroxylated metabolite at the piperidine and at the ethyl linker – Acrylfentanyl	
Butyrfentanyl 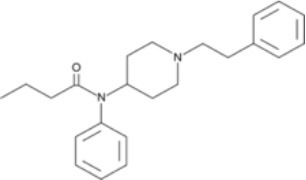	*In vitro*	Human liver microsomes	36	– N-dealkylated metabolite – Hydroxy-metabolite at butanamide chain – Dihydroxy-metabolite at phenylethyl ring			Steuer et al., 2017
	*In vivo*	Urine (human)		– Carboxy-metabolite at butanamide chain – Hydroxy-metabolite at butanamide chain – Carboxy at butanamide chain and hydroxy at phenylethyl ring metabolite	Glucuronides of hydroxy-metabolites	– N-dealkylated metabolite – Hydroxy-metabolite at butanamide chain – Carboxy-metabolite at butanamide chain	
		Blood (human)		– Carboxy-metabolite at butanamide chain			
Carfentanil 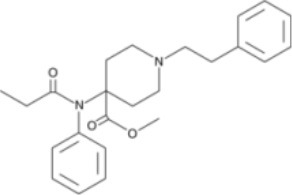	*In vitro*	Pool human liver hepatocytes	11	– Monohydroxylated metabolite at of piperidine ring N-dealkylated metabolite	Glucuronide of hydroxylated metabolite	– Monohydroxylated metabolite at of piperidine ring	Feasel et al., 2016
Furanylfentanyl (Fu-F) 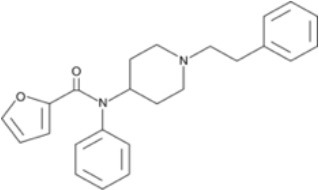	*In vitro*	Human hepatocytes Pooled human hepatocytes	13	– Amide hydrolysis – N-dealkylated metabolite – Dihydrodiol metabolite at furan group			Watanabe et al., 2017
	*In vivo*	Urine (human)	9	– Amide hydrolysis – Dihydrodiol metabolite at furan group – Dihydrodiol at furan group and hydroxy at ethyl linker metabolite	Glucuronide and sulfate of hydroxylated metabolites	– Dihydrodiol metabolite at furan group	
	*In vivo*	Urine (human)		– Amide hydrolysis – Dihydrodiol metabolite at furan group	Sulfate metabolite of amide hydrolysis metabolite	– Sulfate metabolite of amide hydrolysis metabolite – Dihydrodiol metabolite at furan group	Goggin et al., 2017
	*In vitro*	Human liver microsomes	17	– Despropnionyl fentanyl – Monohydroxylated metabolite – N-dealkylated metabolite			Gaulier et al., 2017
	*In vitro*	HepaRG cell Line	17	– Despropnionyl fentanyl – N-dealkylated metabolite – Dihydrodiol metabolite (at furan group)	Glucuronide hydroxylated metabolite		
4-Fluoro-isobutyrylfentanyl 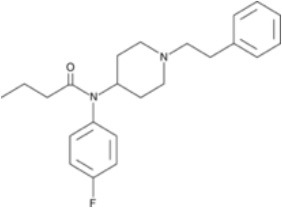	*In vitro*	Pooled human hepatocytes	9	– N-dealkylated metabolite of the piperidine moiety – Monohydroxy metabolite at the piperidine ring or at the ethyl linker – N-oxidation at the piperidine ring			Watanabe et al., 2017
	*In vivo*	Urine (human)	13	– N-dealkylated metabolite of the piperidine moiety – Monohydroxy metabolite at the piperidine ring or at the ethyl linker – Hydroxymethoxy metabolite at phenylethyl ring	Glucuronide hydroxylated metabolites	– Monohydroxy metabolite at the piperidine ring or at the ethyl linker – Hydroxymethoxy metabolite at phenylethyl ring	
Isofentanyl 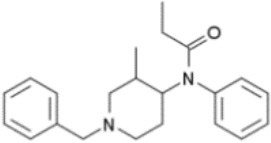	*In vitro*	Urine (rats)	11	– N-dealkylation followed by hydroxylation of the alkyl and aryl moiety – Hydroxylation of the propanamide side chain followed by oxidation to the carboxylic acid – Hydroxylation of the benzyl moiety followed by methylation – N-oxidation	Glucuronides of hydroxy metabolites	– N-dealkylated metabolite	Meyer et al., 2012
3-methylfentanyl 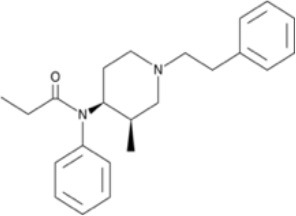	*In vivo*	Urine (rats)	9 /5	– N-dealkylation followed by hydroxylation of the alkyl and aryl moiety – Hydroxylation of the propanamide side chain followed by oxidation to the carboxylic acid – Hydroxylation of the benzyl moiety followed by methylation	Glucuronides of hydroxy metabolites	– N-dealkylated metabolite	Meyer et al., 2012
Ocfentanil (OcF) 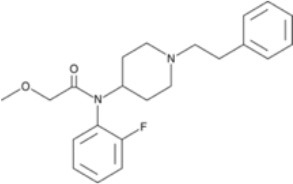	*In vitro*	Human liver microsomes	3	– O-desmethyl metabolite – Monohydroxylated metabolite at phenylethyl ring – O-desmethyl metabolite hydroxylated at phenylethyl ring	Glucuronide of O-desmethylated metabolite		Allibe et al., 2018
	*In vivo*	– Blood (human, *n* = 1) – Bile (human, *n =* 1)	3	– O-desmethyl metabolite – Monohydroxylated metabolite at phenylethyl ring – O-desmethyl metabolite hydroxylated at phenylethyl ring		– O-desmethylated-metabolite	
AH-7921 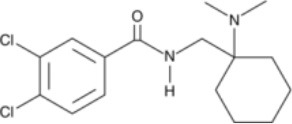	*In vitro*	Human hepatocytes	11	– N-demethyl metabolite – N-dis-demethyl metabolite – N-demethyl metabolite hydroxylated at cyclohexyl	Glucuronide demethylated metabolite		Wohlfarth et al., 2016
	*In vivo*	Urine (human)	10	– N-demethylation – N-dis-demethyl metabolite	Glucuronide demethylated metabolite	– N-demethylation – N-dis-demethyl metabolite	
MT-45 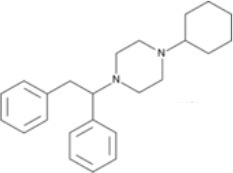	*In vitro*	Rat hepatocytes	10	– Hydroxy metabolite – Dihydroxy metabolite – 1-cyclohexyl-piperazine	Glucuronides of hydroxy metabolites		Montesano et al., 2017
	*In vivo*	Urine (rat)	10	– Hydroxy metabolite – Dihydroxy metabolite – 1-cyclohexyl-piperazine – OH-1-cyclohexyl-piperazine	Glucuronides of hydroxy metabolites	– Hydroxy metabolite – Dihydroxy metabolite	
U-47700 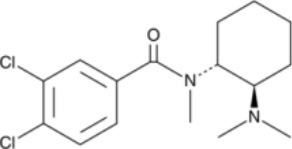	*In vitro*	Human liver microsomes	4	– N-desmethyl-U-47700 – N,N-didesmethyl-U-47700 – N-desmethyl-hydroxy-U-47700 – N,N-didesmethyl-hydroxy-U-47700			Krotulski et al., 2018a
	*In vivo*	Urine (human, *n =* 5)	5	– N-desmethyl-U-47700 – N,N-didesmethyl-U-47700 – N-desmethyl-hydroxy-U-47700 – N,N-didesmethyl-hydroxy-U-47700 – N,N-didesmethyl-N-desmethyl-U-47700		– N-desmethyl-U-47700 – N,N-didesmethyl-U-47700	
U-49900 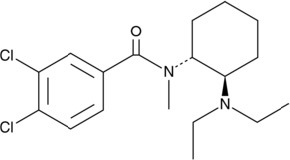	*In vitro*	Human liver microsomes	5	– N-desethyl-U-49900 – N,N-didesethyl-U-49900; – N,N-didesethyl-N-desmethyl-U-49900 – N-desethyl-hydroxyo-U-49900 – N-desethyl-N-desmethyl-U-49900			Krotulski et al., 2018a
	*In vivo*	Urine (human, *n =* 5)	5	– N-desethyl-U-49900 – N,N-didesethyl-U-49900 – N,N-didesethyl-N-desmethyl-U-49900 – N-desethyl-hydroxyo-U-49900 – N-desethyl-N-desmethyl-U-49900		– N,N-didesethyl-N-desmethyl-U-49900	

Fentanyl-derivatives metabolism studies showed similarities and differences from fentanyl metabolism pathways and rates. These different metabolic pathways observed for certain derivatives, demonstrate the need to perform individual metabolism studies for each new compound. In the case of fentanyl, only less than 8% of fentanyl is excreted unchanged. Approximately 85% of the dose is excreted within 72 h in feces and urine, the majority as metabolites mainly as norfentanyl generated by N-dealkylation at the piperidine nitrogen (McClain and Hug, 1980). Minor fentanyl metabolites are despropionylfentanyl, also known as 4-ANPP, which is formed by carboxamide hydrolysis, and hydroxyfentanyl and hydroxynorfentanyl metabolites, both hydroxylated at the propionyl moiety (Goromaru et al., 1984; Mahlke et al., 2014).

Several synthetic opioids follow a similar metabolic pathway to fentanyl. Alfentanil undergoes piperidine N-dealkylation to noralfentanil (Meuldermans et al., 1988). Major alpha-methylfentanyl metabolites in rats were norfentanyl and hydroxypropionyl norfentanyl metabolites, exactly as fentanyl (Sato et al., 2010). Meyer et al. (2012) investigated the metabolism in rats of isofentanyl and 3-methyl fentanyl. After the administration of suspected recreational doses, the parent drugs could not be detected in urine and their common nor-metabolite was the predominant compound.

Patton et al. (2014) detected high concentrations of acetylfentanyl and acetyl norfentanyl (>16,500 ng/mL, 180 min post-dose) in urine samples from rats treated with a toxic dose of acetylfentanyl (3 mg/kg); however, Melent'ev et al. (2015), showed that the main pathway of the biotransformation of acetylfentanyl was hydroxylation by the phenylethyl moiety rather than N-dealkylation in authentic human samples. Melent'ev et al. (2015) and Watanabe et al. (2017) recommended as target analytes in human urine hydroxy-methoxy at phenylethyl moiety and monohydroxylated metabolites, although the reported hydroxylation position in both publications was different. In both publications, the parent compound acetylfentanyl was highly abundant in urine samples, indicating that the parent drug is a suitable target.

Acrylfentanyl underwent N-dealkylation at the piperidine nitrogen producing the major nor-metabolite (Watanabe et al., 2017). The parent compound was also detected at high concentrations in urine samples. N-Dealkylation and monohydroxylation of the piperidine ring were the dominant metabolic pathways for carfentanil *in vitro* (Feasel et al., 2016). In that study, the authors observed a slow parent depletion in the hepatocytes. For 4-fluoroisobutyrylfentanyl the main metabolites identified in urine were the nor-metabolite, and monohydroxy metabolites at the piperidine ring or at the ethyl linker, as well as the parent compound. In terms of specificity, Watanabe et al., recommended as target compounds in urine the monohydroxy metabolites and the hydroxymethoxy metabolite (Watanabe et al., 2017).

In the case of butyrfentanyl, hydroxylation of the butanamide side chain followed by subsequent oxidation to the carboxylic acid represented the major metabolic step (Steuer et al., 2017). Although the norbutyrfentanyl was not among the most abundant metabolites in human samples in that study, the authors suggested its inclusion as a recommended target analyte because it showed a high intensity in the *in vitro* experiment. In authentic postmortem blood and urine samples, butyrfentanyl was still detected at 66 and 1,000 ng/mL, respectively.

Furanylfentanyl contains a furan group that affects its metabolic profile. This structure seemed to favor the amide hydrolysis, which is the main metabolite *in vitro* and *in vivo* (Watanabe et al., 2017). In terms of specificity of the target metabolites, Watanabe et al. (2017) recommended the dihydrodiol-metabolite and Goggin et al. (2017) recommended the same metabolite, as well as the sulfate of the metabolite that results from the amide hydrolysis. As it happened with butyrfentanyl (Steuer et al., 2017), the hepatocyte experiment also suggested high prevalence for the nor-metabolite, which was not significantly present in the authentic urine samples, illustrating the need to analyze human specimens. Furanylfentanyl parent compound was detected in authentic urine samples. For ocfentanyl, the predominant metabolite detected in blood, along with the parent drug, was the O-desmethylated metabolite (Allibe et al., 2018).

In the case of the new synthetic opioids not structurally related to fentanyl, different metabolic pathways has been reported. For AH-7921, the preferred metabolic sites were the amine function and the cyclohexyl ring. The two most dominant metabolites after hepatocyte incubation (also identified in a urine case specimen) were desmethyl and di-desmethyl AH-7921. Together with the glucuronidated metabolites, they were recommended as suitable analytical targets for documenting AH-7921 intake (Wohlfarth et al., 2016). In the case of MT-45, Montesano et al reported hydroxy-MT-45-glucuronide and di-hydroxy-MT-45-glucuronide as the most abundant metabolites in rat urine, while the parent drug was found at concentrations <10 ng/mL after 300 min (Montesano et al., 2017). Although similar in chemical structure, U-47700 and U-49900 showed specific metabolites. N-Desmethyl-U-47700 was identified as the major metabolite in human urine specimens, and N,N-Didesethyl-N-desmethyl-U-49900 was identified as the most abundant metabolite present. Unlike U-47700 specimens, U-49900 was detected in low abundance in urine samples (Krotulski et al., 2018a).

As indicated by Watanabe et al. (2017), the target metabolites should generally be abundant, specific of the parent drug, and prevalent in most, if not all, case samples. Given the strong structural similarities among emerging designer fentanyls, many of them are coincidentally biotransformed to the exact same metabolite. This fact can make identification of the specific parent drug in a case difficult. The ability to identify minor metabolites that are unique and specific to the parent drug is therefore of considerable importance. 4-ANPP can be formed by fentanyl and other different fentanyl analogs metabolism, and it is also a precursor contaminant found in seized illicit fentanyl and analogs, so its presence is not particularly diagnostic. Other common metabolites are: acetylnorfentanyl from acetyl-alpha-methylfentanyl or acetylfentanyl (Watanabe et al., 2017); norfentanyl from fentanyl, beta-hydroxythiofentanyl and alpha-methyl-fentanyl (Sato et al., 2010); norcarfentanil from carfentanil, sufentanil and remifentanil (Feasel et al., 2016). 3,4-dichloro-N-(2-aminocyclohexyl)-N-methyl-benzamide is a common metabolite of U-47700 and U-49900, but it is not a major metabolite in urine for either compound (Krotulski et al., 2018a).

Another important aspect of the metabolism is the identification of the enzymes involved. Pharmacokinetic interactions may be produced due to the presence of other substances metabolized by the same enzymes, ultimately affecting the drug blood concentrations. Fentanyl, sufentanyl and alfentanil are mainly metabolized by CYP 3A4 (Feierman and Lasker, 1996; Guitton et al., 1997). Steuer et al., identified CYP 3A4 and CYP 2D6 as the isoforms involved in the metabolism of butyrfentanyl (Steuer et al., 2017). Meyer et al., reported that CYP 3A4, CYP 3A5 and CYP 2C19 are involved in the metabolism of 3-methylfentanyl and isofentanyl and, in the case of isofentanyl, additionally CYP2D6 (Meyer et al., 2012). Remifentanil is the only family member of this class found to be ~95% metabolized in the blood and tissues by non-CYP enzymes, probably due to an easily accessible ester group allowing rapid hydrolysis by circulating blood esterases (Bürkle et al., 1996).

## Concentrations in postmortem specimens and other findings

The concentrations determined in postmortem specimens varied considerably depending on the type of synthetic opioid detected. Derivatives with potencies relative to morphine of more than 170, showed concentrations in femoral blood in the low ng/mL or pg/mL range, while those derivatives with potencies similar to morphine showed concentrations of hundreds, and even thousands, of ng/mL. An exception happens with furanyl fentanyl, which is seven times more potent than morphine (Higashikawa and Suzuki, 2008), but the reported femoral concentrations were less than 50 ng/mL. Typical morphine postmortem concentrations in blood in fatalities are from 200 to 2,300 ng/mL, for methadone 400 to 1,800 ng/mL, for buprenorphine 1.1–29 ng/mL and norbuprenorphine (active metabolite) 0.2–13 ng/mL (Baselt, 2017), and for oxymorphone 23–554 ng/mL (Crum et al., 2013). The potency of the different drugs affects their lethal levels, but other important issues, such as the presence of other CNS depressant drugs, and developed opioids tolerance, have to be taken into account in the interpretation of the concentrations. The derivative with the highest number of published cases was acrylfetanyl, and with the lowest MT-45. Table 4 summarizes the concentrations of the parent drugs found in case reports and articles where overdose due to a specific opioid was the cause of death.

**Table 4:** Postmortem concentrations in different biological samples for synthetic opioids (median, range, number of cases).

**Analyte**	**Blood (ng/mL)**				**Vitreous humor (ng/mL)**	**Brain (ng/g)**	**Liver (ng/g)**	**Urine (ng/mL)**
	**Femoral**	**Cardiac**	**Subclavian**	**Non-specified**				
3-Methylfentanyl	–	–	–	0.4 (0.3–0.9) *n =* 3	–	–	–	–
4–fluorobutyr fentanyl	–	–	–	91–112 *n =* 2	–	248 *n =* 1	902 *n =* 1	200 *n =* 1
Acetylfentanyl	223.5 (16–600) *n =* 12	270 (170–2,100) *n =* 11	220 *n =* 1	–	140–240 *n =* 2	620 *n =* 1	1,000–1,100 *n =* 2	2,660 (240–3,420) *n =* 4
Acrylfentanyl	0.2 (0.01–5) *n =* 42	–	–	–	–	–	–	–
Butyryl fentanyl	99 (66–145.2) *n =* 3	60.5 (39–220) *n =* 3	–	–	32 *n =* 1	93–200 *n =* 2	41–57 *n =* 2	64 *n =* 1
Carfentanil	0.2 (0.01–0.5) *n =* 9	0.1–0.2 *n =* 2	0.03 *n =* 1	–	–	–	–	–
Fentanyl	11 (1–60) *n =* 207	13 (1.8–139) *n =* 81	–	13 (2–383) *n =* 66	14.8 (8–20) *n =* 4	49 *n =* 1	78 (5.8–16,983) *n =* 99	97 (2.9–1,200) *n =* 31
Furanyl fentanyl	2.7 (0.4–42.9) *n =* 13	2.8 *n =* 1	–	–	–	–	–	–
Ocfentanyl	9.1 (3.7–15.3) *n =* 3	23.3 (3.9–27.9) n3	–	–	12.5 *n =* 1	37.9 *n =* 1	31.2 *n =* 1	6–480 *n =* 2
AH–7921	350 (30–9,100) *n =* 13	480–3,900 *n =* 2	–	–	190 *n =* 1	7,700 *n =* 1	530–26,000 *n =* 2	760–6,000 *n =* 2
MT–45	520–660 *n =* 2	1,300 *n =* 1	–	–	260 *n =* 1	–	24,000 *n =* 1	370 *n =* 1
U–47700	358 (189–1,460) *n =* 12	691.5 (260–1,347) *n =* 4	–	–	130 (90–170) *n =* 2	(0.9–380) *n =* 3	142.1 (3.1–1,700) *n =* 4	1620.5 (360–4,600) *n =* 4

*3-Methylfentanyl references: (Ojanperä et al., 2006)*.

*4-fluorobutyr fentanyl references: (Rojkiewicz et al., 2017)*.

*Acetylfentanyl references: (Pearson et al., 2015; Poklis et al., 2015; Cunningham et al., 2016; Fort et al., 2016; McIntyre et al., 2016a; Takase et al., 2016; Yonemitsu et al., 2016; Dwyer et al., 2018)*.

*Acrylfentanyl references: (Butler et al., 2017; Guerrieri et al., 2017b)*.

*Butyryl fentanyl references: (Poklis et al., 2016; Staeheli et al., 2016)*.

*Carfentanil references: (Shanks and Behonick, 2017; Swanson et al., 2017; Hikin et al., 2018)*.

*Fentanyl references: (Anderson and Muto, 2000; Kuhlman et al., 2003; Martin et al., 2006; Coopman et al., 2007; Biedrzycki et al., 2009; Carson et al., 2010; Krinsky et al., 2011, 2014; Palamalai et al., 2013; Marinetti and Ehlers, 2014; McIntyre et al., 2014; Bakovic et al., 2015; Moore et al., 2015; Pearson et al., 2015; Poklis et al., 2015; Rodda et al., 2017; Dwyer et al., 2018)*.

*Furanyl fentanyl references: (Mohr et al., 2016; Guerrieri et al., 2017a; Martucci et al., 2017; Papsun et al., 2017)*.

*Ocfentanyl references: (Coopman et al., 2016; Dussy et al., 2016; Allibe et al., 2018)*.

*AH-7921 references: (Karinen et al., 2014; Kronstrand et al., 2014; Vorce et al., 2014; Fels et al., 2017)*.

*MT-45 references: (Papsun et al., 2016; Fels et al., 2017)*.

*U-47700 references: (Elliott et al., 2016; Mohr et al., 2016; Dziadosz et al., 2017; Papsun et al., 2017; Rohrig et al., 2017)*.

In several cases, multiple synthetic opioids were detected. Acetylfentanyl and fentanyl were frequently found together (Pearson et al., 2015; Poklis et al., 2015; Dwyer et al., 2018). Other combinations were butyryl fentanyl and acetyl fentanyl (McIntyre et al., 2016b; Poklis et al., 2016), or U-47700 (Mohr et al., 2016); furanyl fentanyl and acetyl fentanyl (Papsun et al., 2017), acryl fentanyl (Butler et al., 2017), butyryl fentanyl (Mohr et al., 2016), fentanyl (Guerrieri et al., 2017a), or carfentanil (Shanks and Behonick, 2017); carfentanil and fentanyl (Shanks and Behonick, 2017); and tetrahydrofuran fentanyl and U-49900 (Krotulski et al., 2018b). The femoral concentrations reported in those combination cases were frequently below the range of the concentrations summarized in Table 4. Acetylfentanyl median and concentration range in multiple synthetic opioids cases were 9.4, 0.4–240 ng/mL (*n* = 15); acrylfentanyl 0.3 ng/mL (*n* = 1); butyrfentanyl 14.9, 0.3–58 ng/mL (*n* = 4); carfentanil 0.08, 0.05–0.1 ng/mL (*n* = 2); fentanyl 8.2, 1.1–38 ng/mL (*n* = 14); furanyl fentanyl 1.7, 0.6–6.1 ng/mL (*n* = 4) and U-47700 17 ng/mL (*n* = 1).

In all of the reports mentioned in Table 4 and above, synthetic opioids were commonly detected with other drugs, especially other CNS depressants, such as benzodiazepines, ethanol and other opioids. This combination may produce a pharmacodynamic interactions and increase the risk of respiratory depression. This possible interaction between opioids, alcohol and benzodiazepines has been previously described for other opioids, such as buprenorphine (Häkkinen et al., 2012; Seldén et al., 2012), methadone (Jones et al., 2012; Pilgrim et al., 2013; Nielsen et al., 2015), oxycodone (Ogle et al., 2012), and heroin (Thaulow et al., 2014). Among the reviewed cases positive for synthetic opioids other than fentanyl, 44 reported as cause of death intoxication due to multiple drugs and 77 intoxication mainly due to one specific opioid. The manner of death was predominantly accidental (*n* = 99), and suicides were reported in 7 cases.

## Postmortem redistribution and stability

Postmortem changes in drug concentrations can happen via postmortem redistribution (PMR) from tissues of a higher to a lower concentration. Physicochemical and pharmacological properties of the analytes, such as pKa, log P, volume of distribution (Vd) and protein binding, may indicate drugs that experience this postmortem phenomenon. Lipophilic basic drugs with a Vd > 3 L/kg, such as fentanyl, may undergo PMR. Fentanyl has been reported to undergo extensive PMR (Luckenbill et al., 2008; Olson et al., 2010; Palamalai et al., 2013; Brockbals et al., 2018). In the case of the synthetic opioids, limited data is currently available about PMR, and as well as information about pKa, log P and Vd (Tables 2, 3). Staeheli et al. (2016) reported postmortem concentration changes of butyrfentanyl and metabolites, suggesting these compounds were prone to PMR. PMR reports about other synthetic opioids are not currently available.

Based on currently published case reports and articles, the cardiac blood-to-femoral blood and liver-to-femoral blood ratios were calculated to predict candidates of PMR. Results are summarized in Table 5. Due to the scarce amount of data available (1–4 cases per analyte), no conclusions could be drawn. Synthetic opioids showed median cardiac-to-femoral ratios around 1, and a tendency to accumulate in the liver. Regarding the distribution to vitreous humor, it may be slow showing higher concentrations in blood. Other factors, such as time of death and sample collection, or rapid vs. delayed deaths, has not been taken into account in this analysis due to the limited data available.

**Table 5:** Postmortem concentration ratios in different biological samples for synthetic opioids (median, range, number of cases).

**Analyte**	**Cardiac-to-femoral**	**Liver-to-femoral**	**Vitreous humor-to-femoral**	**References**
Acetylfentanyl	1.2 (0.8–1.6) *n =* 4	3.8–5.7 *n =* 2	0.6–0.9 *n =* 2	Cunningham et al., 2016; Fort et al., 2016; McIntyre et al., 2016a; Yonemitsu et al., 2016
Butyryl fentanyl	0.6 (0.4–2.2) *n =* 3	0.4–0.9 *n =* 2	0.3 *n =* 1	Poklis et al., 2016; Staeheli et al., 2016
Fentanyl	(0.7–4.6) *n =* 54	6.6 (1.4–539.4) *n =* 75	1.5 (1.1–1.8) *n =* 3	Anderson and Muto, 2000; Krinsky et al., 2011, 2014; Palamalai et al., 2013; McIntyre et al., 2014; Bakovic et al., 2015
Furanyl fentanyl	1.5 *n =* 1	–	–	Martucci et al., 2017
Ocfentanyl	1.5 (1.1–3.1) *n =* 3	2 *n =* 1	0.8 *n =* 1	Coopman et al., 2016; Dussy et al., 2016; Allibe et al., 2018
AH–7921	0.4–1.1 *n =* 2	1.2–2.9 *n =* 2	0.4 *n =* 1	Vorce et al., 2014; Fels et al., 2017
MT-45	2 *n =* 1	36.4 *n =* 1	0.4 *n =* 1	Fels et al., 2017
U-47700	1.5 (0.7–2.6) *n =* 4	0.4 (0.003–8.9) *n =* 4	0.2–0.9 *n =* 2	Dziadosz et al., 2017; Rohrig et al., 2017

PMR is still a controversial issue for classic opioids. Hargrove and Molina (2014) showed insignificant redistribution of morphine from central sites within 24 h after death in bodies kept at 4°C, while Staeheli et al. (2017) observed a significant increase of morphine concentration, although these changes were not relevant for forensic interpretation. Morphine-derivatives, such us hydrocodone (Saitman et al., 2015), codeine (Frost et al., 2016), and oxycodone (Brockbals et al., 2018), are unlikely to undergo substantial PMR changes. More lipophilic opioids with higher Vd, like methadone (Jantos and Skopp, 2013; Holm and Linnet, 2015; Brockbals et al., 2018), may undergo PMR.

Several studies have been conducted to evaluate stability of fentanyl and some of its derivatives in fortified biological samples, such as blood, plasma and urine. Eleven fentanils (fentanyl, norfentanyl, carfentanil, norcarfentanil, sufentanil, norsufentanil, lofentanil, 3-methylfentanyl, alfa-methylfentanyl, ohmefentanyl, and remifentanil acid metabolite), were stable in urine samples stored at −20°C or below for at least 2 months. However, remifentanil in urine samples decreased by approximately 90% within 1 week at room temperature and by more than 50% in samples stored for 1 week at 4°C. Because of the instability of that analyte, the authors recommended to analyze the primary metabolite, remifentanil acid (Wang and Bernert, 2006). Fentanyl and its metabolites norfentanyl, despropionylfentanyl and hydroxynorfentanyl were stable in urine after 3 freeze-thaw cycles, and after storage at −20°C for 2 months (Mahlke et al., 2014).

Fentanyl, norfentanyl, acetyl fentanyl and acetyl norfentanyl spiked into whole blood were stable after three freeze-thaw cycles and at room temperature for 72 h (Poklis et al., 2015). No loss of fentanyl concentration could be observed after 3 months of storage at 4–8°C and −20°C in blood samples at 5 and 10 ng/mL (Andresen et al., 2012). However, another study showed fentanyl and its metabolites norfentanyl, despropionylfentanyl and hydroxynorfentanyl lose up to 51.6% after 3 freeze-thaw cycles, and fentanyl and despropionylfentanyl up to 34.8% after storage at −20°C for 2 months (Mahlke et al., 2014). Furanylfentanyl showed no significant degradation in blood samples at 5 and 10 ng/mL 48 h room temp and at 4°C 7 days (Guerrieri et al., 2017a) and up to 30 days (Mohr et al., 2016).

Regarding the new synthetic opioids not related to fentanyl, U-47700 was stable in blood refrigerated for up to 30 days (Mohr et al., 2016). AH-7921 was found to be stable for at least 21 days in blood and plasma at room temperature (Soh and Elliot, 2014). In the case of MT-45, a loss of 50% was observed after 12 months of storage (Papsun et al., 2016). Further studies are necessary to evaluate the stability of the different synthetic opioids and metabolites, and in additional biological samples of forensic interest, such as vitreous humor and tissues.

## Conclusion

We performed a critical review of the currently available literature to assist in the toxicological interpretation of synthetic opioids postmortem cases. Synthetic opioids constitute a heterogenous group of compounds related or not to fentanyl, mostly basic and lipophilic, with a wide range of potencies related to morphine, from 1 to 10,000. Research has been conducted in the investigation of metabolic pathways and identification of target metabolites of fentanyl derivatives and non-structurally related synthetic opioids, showing similarities and differences from fentanyl depending on the compound. Postmortem concentrations seemed to correlate with their potency, although the presence of other CNS depressants, such as ethanol and benzodiazepines has to be taken into account. Further research is guaranteed to investigate postmortem redistribution phenomena of this class of compounds, and stability issues in postmortem samples.

## Author contributions

MC and GC contributed conception and design of the review. MC, RC, and JP searched, organized, reviewed and analyzed the case reports and research articles. MC wrote the first draft of the manuscript. All authors contributed to manuscript revision, read and approved the submitted version.

### Conflict of interest statement

The authors declare that the research was conducted in the absence of any commercial or financial relationships that could be construed as a potential conflict of interest.
